# Fake News through the Eyes of Three Generations of Russians: Differences and Similarities in Social Representations^1^

**DOI:** 10.11621/pir.2022.0106

**Published:** 2022-03-30

**Authors:** Alexander Sh. Tkhostov, Alexander M. Rikel, Margarita Ye. Vialkova

**Affiliations:** a Lomonosov Moscow State University, Moscow, Russia

**Keywords:** generation, social representations, fake news, media trust, Generation Z, Millennials

## Abstract

**Background:**

The problem of fake news becomes especially prominent during periods of social exacerbation, such as the coronavirus pandemic, wherein the events have a significant impact on many lives. Generational differences are considered as a factor affecting perceptions of the reliability of news.

**Objective:**

The aim of this study was to reveal and compare the social representations of information reliability and news verification criteria among people belonging to the Generation of Reforms (born 1968-1981), the Millennial Generation (1982-2000) and Generation Z (2001 and later) in Russia.

**Design:**

The study involved 431 participants and was comprised of two stages: focus groups and a survey. The data analysis methods employed were thematic analysis, qualitative and quantitative content analysis, coefficient of positive answers (according to J. Abric), Kruskal-Wallis H test, Pearson’s chi-square test, Spearman’s rank correlation coefficient, and Kendall’s t-rank correlation coefficient.

**Results:**

We have found significant differences between the Generation of Reforms (CPA: 80,5; p = 0,000) and Generation Z (CPA: 90,2; p = 0,000), and similarities between the Millennial Generation (CPA: 90,3; p = 0,000) and Generation Z, in the structure and content of social representations regarding “fakes”. Notably, Generation Z favors a fact-checking strategy to identify news reliability, while “Reformists” rely on offline contacts.

**Conclusion:**

Generations in Russia differ with respect to their tolerance of “fakes” and their strategies for news verification. The results advance our understanding of “fakes” as purely social constructs. The attribution of media incompetence to older and younger cohorts by each other was discussed as the generational conflict.

## Introduction

Our time is often referred to as the “post-truth era”. Modern-day people rely on emotional factors as opposed to facts, and turn to the mass media for verification of opinions. Thus, the *reliability* of information becomes uncertain (Tulupov, 2020; McIntyre, 2018; d’Ancona, 2017). There is a paradox that describes information consumption today: blind trust leads to deception, while distrust paralyses one’s ability to choose between many sources that appear unreliable. In such circumstances, a person’s capacity to think critically and discern so-called *“fakes”* decreases.

Fake news is rarely identified due to the following:

1. A disinclination to abandon beliefs and attitudes that are encouraged by fake news.
2. Personal rigidity and coping strategies that exist to maintain an accustomed order and image of the world.
3. A lack of competence or knowledge of the need for fact-checking (i.e. verification of information for accuracy) (Barthel et al., 2016)
4. The effects of “information warfare”, which is made up of fake news and mutual accusations of the production of “fakes”.

Thereby, “fakes” have become a classic example of a social construct (Raskin, 2006). A “fake” could be identified by the expert community, but this is almost unattainable in everyday discourse, due to the perceptual replacement of facts with *media facts* (media content that a person considers to be fact) (Beloyedova, Kazak 2017; Morozova, 2017; Prom, 2020). Identification of fake news is a complex activity requiring certain competencies and skills. At the same time, it is necessary to understand that in several cases where in-depth expert analysis was employed, a perfect method to distinguish between “fakes” and “non-fakes” was not found. Therefore, even critical-minded specialists are forced to stop at some iteration of fact-checking.

In this study, we define fake news as completely or partially false information, *deliberately* presented as veracious news to mislead the audience (Kalsnes, 2018). Fake news can spread rapidly, as authors use techniques to manipulate people’s emotions, capture attention and arouse interest, trust and desire to share the information (Yershov, 2018; Zelinskiy, 2018). In particular, the COVID-19 pandemic has generated nationally specific “fakes’’ that depend on the cultural environment and level of technological development (Sadykov, Akhmetianova, 2020).

The crucial difference between modern “fake news” and old “newspaper ducks” lies in its circulation: information can now be disseminated through reposts easily, quickly, and extremely widely. The issue of the appearance of fake news is aggravated by the reaction to it, leading to decreased *trust* in mass media (Nazarov et al., 2019) and likely contributing to the culture of low trust in public institutions in Russia. Trust in mass media is based on the recent understanding of mass media as a social media, which implies a reciprocal process of transferring information, communication, and feedback (Dukin, 2016; Nenasheva, 2018; Thornley, 2008). According to the latest trends, people consider the internet to be a reliable a source of information, while they perceive television as a form of entertainment and attribute negative features to it i.e., mental disruption and mass consciousness manipulation. Similar results can be found in sociological surveys (Zhizhina, 2018; Efanov, 2018).

**Figure 1.**
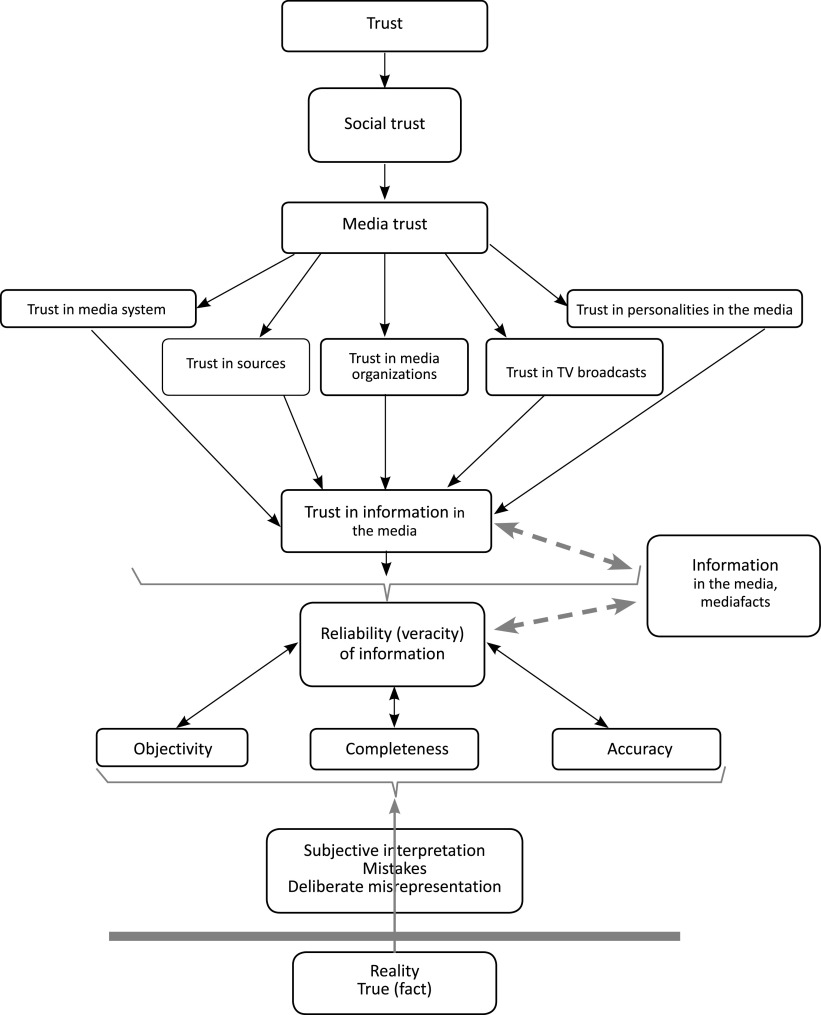
Key concepts of trust in information.

*Note. The scheme illustrates the relationships between concepts in our study*.

In *Figure 1*, we present the key concepts in our study (truth, trust, trust in the media, trust in information, reliability of information) and the relations between them. Trust in the media is associated with personal and situational factors, values, emotional and motivational domain, and peculiarities of thinking and behavior (Shanin, 2018). Special attention is paid to the four information types presented in the media: 1) business, practically useful, 2) business, practically useless, 3) entertaining, practically useful, 4) entertaining, practically useless (Kupreychenko, Shlyakhovaya, 2012). In this report , we focus on generational differences as a factor affecting social representations of the reliability of information. We consider generations in the socio-psychological paradigm as contemporaries i.e., people who may be of different ages, but experience the same events and historical periods at the same time (Kon, 1978; Sadykova, 2015; Mannheim, 1970; Isaeva, 2011b).

Recent intergenerational studies conducted abroad on this subject have shown interesting results. An experimental study implying the publication of fake news concerning Brexit on Facebook found that most people who took headlines at face value without clicking, belonged to older generations (Loos, Nijenhuis, 2020). On the other hand, D.Trninic and colleagues from Bosnia and Herzegovina have shown that younger generations also lack competence when it comes to fake media fact-checking. They have also shown that promoting education in the social media sphere might bring about a long-term solution for the battle against “fake news” (Trninic et al., 2022). The problem seems to also be relevant to children of elementary school age (Pilgrim, Vasinda, 2021).

In our research, we classify the generations to be investigated based on the works of Y. Levada (2011) modified by V.V. Radaev (2020): the Generation of Reforms (born 1968-1981), the Millennial Generation (1982-2000) and Generation Z (2001 and later). Consistent with previous research, we use this particular classification due to its adaptation to the Russian social context and ongoing events, as well as its significant empirical validity (Radaev, 2020). The study involves representatives of these three cohorts that frequently consume media information. Generation Z has previously been found to possess greater information surfing abilities (Soldatova, Chigarkova, Rasskazova, 2020), hence our assumption that this generation might be more tolerant towards fake news and more competent in identifying “fakes”.

The *object* of this study is the evaluation of information reliability in the media and on social media. The *subject* is social representations of information reliability in the mass media and on social media among people belonging to different generations. The *goal* is to reveal the structure and content of social representations regarding the reliability of information in the mass media and on social media among people belonging to the Generation of Reforms, the Millennial Generation and Generation Z. We *hypothesize* that there are differences between the Generation of Reforms, the Millennial Generation and Generation Z in the structure and content of social representations of the criteria for evaluating the reliability of news in the mass media and on social media.

## Methods

### Participants

The pilot stage of the study (focus groups) involved 31 participants (women — 21 (68%), men — 10 (32%); 10 people from Generation Z (over 17 years old), 11 people from the Millennial Generation, 10 people from the Generation of Reforms). 29 participants were from Moscow or the Moscow region; most of the respondents possessed higher education qualifications or were in the process of obtaining them, the rest were in upper secondary education. We held 9 focus groups, three for each generation. The second stage of the study (survey) involved 400 respondents (women — 297 (74.2%), men — 103 (25.8%); 123 (30.8%) people from Generation Z, 154 (38.5%) people from the Millennial Generation, 123 (30%) people from the Generation of Reforms). More than 60% of the participants were from Moscow, and less than 40% were from other regions. 236 respondents (60%) possessed higher education qualifications, 103 (25.8%) were in the process of obtaining higher education qualifications, 19 (4.8%) had been through specialized secondary education, 30 (7.5%) had been through secondary education, and 12 (3%) were in the process of completing their secondary education.

### Procedure

Data collection methods: focus group, survey (online). Data analysis methods: thematic analysis, qualitative and quantitative content analysis, coefficient of positive answers (according to J. Abric), Kruskal-Wallis H test, Pearson’s chi-square test, Spearman’s rank correlation coefficient, Kendall’s t-rank correlation coefficient.

Questionnaires: (1) “Interpersonal Trust Scale” by J. Rotter (levels of social and institutional trust) (Leonova, Leonov, 2016); (2) “Faith in People” by M. Rosenberg (level of basic trust) (Robinson et al., 2013); (3) The authors’ questionnaire, based on the results of the pilot stage of the study (focus groups) — the structure and content of social representations regarding the reliability of news in the mass media and on social media.

Our questionnaire includes: (3а) Demographic questions, questions regarding preferred news sources and an estimation of the likelihood of spread of reliable and fake news within these sources, open-ended questions about the concept of “fake news” and how respondents interpret it; (3b) Seven blocks of statements (total: 105): 1) the evaluation of fake news presence in news sources, 2) reasons for fake news presence, 3) the person’s interaction with fake news, 4) criteria for the identification of the reliability of news, 5) an assessment of people from other generations, 6) an evaluation of the scope of false information spread in the past, present and forecast for the future, 7) possible actions to solve the problem of fake news.

We based this study on the concept of social representations by S. Moskoviсi, in order to investigate “fakes” within social constructionism (Molliner, Bovina, 2020; Molliner, Bovina, 2021). S. Moscovici defines social representation as “a set of concepts, beliefs and explanations that emerge in everyday life in the process of interpersonal communications”. He also calls it “a modern version of common sense” (Emelyanova, 2001). “Reliability of information” is studied as an object of social representations. Firstly, this is because it is considered part of the widespread discourse within society (Ershov, 2018; Kolesnichenko, 2018). Secondly, when people are confronted with new information, particularly news (both reliable and fake), they develop a need to establish a stance on this new knowledge and to find a place for it within their existing belief systems. This relates information in the mass media to the criteria of reliability, which contributes to the formation of social representations. Thirdly, the structure and content of social representations is understood clearly by researchers in times of social crises and upheavals such as the COVID-19 pandemic (Levada, 2021).

In this study, we apply the structural approach of J. Abric (Abric, 2000). The approach involves allocation of the core and peripheral system. The core is associated with concepts of collective memory i.e., the common historical experience of the group. It is stable and its cognitive elements are shared by the whole group, providing homogeneity and generating the meaning of the social representation. The peripheral system is more sensitive to context and not as stable as the core. It takes into account individual experience and distinct features of each group member and plays a part in adaptation to environmental changes (Abric, 1993; Emelyanova, 2001). To identify the structure of social representation, we use the Vergés prototypical analysis method (Vergés, 1992; Bovina, 2011) and the method for calculating the coefficient of positive answers (hereinafter CPA) by J. Abric (TCP — Tauxcatégoriquepositif), adapted by T.P. Emelyanova (Abric, 2000; Emelyanova, 2015; Emelyanova, Schmidt, 2021). This approach allows us to highlight the similarities and differences between the three generations, and to obtain a holistic picture of the social representations for the various thematic blocks identified within focus groups and the survey.

## Results

Across the two stages of the study, we reveal the generation-dependent social representations of information (news) reliability in the mass media and on social media and of the problem of fake news. The results of each stage are presented below in blocks: they complement each other and allow us to compare representations between different generations, as well as between people with varying levels of trust.

### What is “reliable information” and “fake news”?

**Figure 2.**
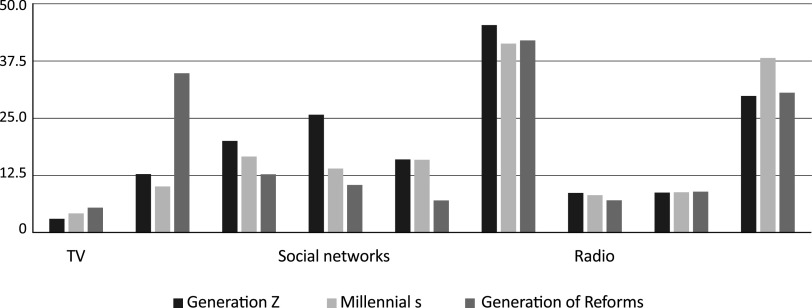
News sources with a high percentage of reliable news (according to participants).

*Note. The histogram illustrates the representations of three generational groups regarding reliable sources of news*.

Consistent with the thematic analysis, participants interpreted reliable information as information that corresponds to reality, correctly and completely reflects real events and facts, excludes distortion during transmission, and has confirmation that it was accurately conveyed. Accuracy of information should be verified by professional journalists. Fake news *was interpreted* by participants as completely or partially false and misleading information, which may contain deliberate or accidental misrepresentation of information about reality. Authors create fake news with the aim of manipulating people’s consciousness, deceiving for some goal, and increasing the popularity of the author. Fake news can be *recognized* by loud headlines, eye-catching words, and clickbait. When compared with a similar social phenomenon — rumors, “fakes” were understood as a more modern, digitized counterpart. Participants evaluated news *sources* by the number of fake vs. reliable news items they contain. Within the three *generational groups,* a large proportion of people (see *Figure 2*) chose “news sites” as a news source with a high percentage of reliable news. However, the second most popular category of choice was “none of the above”, indicating that the respondents tend to consider few sources of information as reliable. The differences between federal television channel ratings given by different generations is also pronounced (see *Table 1*). The Generation of Reforms rated *federal channels* significantly higher than the Millennial Generation and Generation Z in terms information reliability (Chi-square, p = < 0.00). At the same time, Generation Z (after “Millennials”) rated *social networks* higher in terms of news reliability (Chi-square, p = <0.14).

**Table 1:** The core elements of social representations for the block “evaluation of fake news presence in news sources”.

Statements	Generation Z (CPA)^a^	Millennials (CPA)	“Reformists” (CPA)
Statements included in the cores of all generations			
On federal channels, news is often presented from a certain position to shape the opinion of the population	93.5	100.0	95.1
News broadcasts on federal channels often hide facts	91.9	91.6	84.6
In current Russian media and social media, there is a large percentage of misrepresentation of news, partial inaccuracy	91.1	96.1	83.7
Fake news is published in those sources that are popular with people	75.6	76.6	78.0
Statements included in the cores of 1-2 generations			
I try not to watch the news on TV, as I think that there is a lot of unreliable news	72.4	77.9	–
There is a large percentage of fake news in modern Russian media and on social media	72.4	73.4	–

*Note. ^a^CPA= the coefficient of positive answers by J. Abric*.

*Messengers* were rated significantly higher (Chi-square, p = <0.03) by Generation Z and “Millennials” as sources with a large percentage of reliable information. The Millennial Generation and Generation Z placed “television in general” first in the fake news the category followed by “federal channels” in second place. The percentage of people who agreed with this assignment from the Generation of Reforms was less. Those from the Generation of Reforms were less likely to agree with the assumption that facts are often withheld on federal channels, and less prone to avoid watching news on federal channels, than people from younger generations.

### How are other generations affected by fake news?

Upon assessment of the generations according to their views on the susceptibility of other generations to trusting fake news, all generations demonstrated a high level of agreement (CPA 80.5, 87.8 and 92.2) that people over 60 and between 51-60 tend to trust fake information, although the Generation of Reforms gave lower scores on this item than the other two generations (see *Table 2*). Generation Z scored people between the ages 41-50 significantly higher (CPA 61.0, p = 0.00) than the Generation of Reforms did. It is also typical that the Generation of Reforms rated the 17–20 age group significantly higher than the younger generations did (CPA 60.2, p = 0.00). Thus, there is a general tendency to estimate that people who are younger or older than you are credulous towards fake news, although such an assessment is not supported by facts.

**Table 2:** The core elements of social representations for the block “assessment of people from other generations”

Statements	Generation Z (CPA)^a^	Millennials (CPA)	“Reformists” (CPA)
Statements included in the cores of all generations			
I think that people over 60 are very susceptible to trusting fake news	87.8	92.2	80.5
I think that people 51–60 are very susceptible to trusting fake news	81.3	87.0	56.9
Statements, included in the cores of 1–2 generations			
I think that people younger than 17 are very susceptible to trusting fake news	–	–	60.2 /
I think that people 41–50 are very susceptible to trusting fake news	61.0	–	–

*Note. ^a^CPA= the coefficient of positive answers by J. Abric*.

### What are the reasons for the spread of fake news?

Participants associated fake news presence with the malicious dissemination of false information by those who strive for power (political or informational) or quick money trying to increase their popularity. Participants from all three generations supposed that fake news is one of the weapons used in information warfare. However, according to participants, fake news is published not only purposefully by professionals but also due to the mistakes and incompetence of journalists (this was considered more by the younger generations, CPA: 67.5, 68.3, compared to the Generation of Reforms — 43.9, p = 0.006, p = 0.000). The spread of fake news is also related to technological development and an increase in the number of news sources. During the focus group stage, participants reflected that globalization and increasing individualism in society can influence the spread of fake news; participants also noted that, in the history of Russia, fake news is a common and deep-rooted phenomenon.

The core of the social representations of Generation Z contains an element of understanding of fake news as a phenomenon native to society (CPA: 74 for Generation Z, while for “Millennials” 53.9, p = 0.000 and for “Reformists’ 59.3, p = 0.009). These findings can be explained by the assumption that Generation Z actively interacts with various information sources from earlier years. Thereby, the phenomenon of fake news becomes habitual and commonplace, just as information misrepresentation in the mass media and on social media, in fact, is.

### Where does fake news fit into my own life?

Many participants reported that they are faced with fake news in their lives and make efforts to filter reliable information sources from unreliable ones. For instance, many respondents during the coronavirus pandemic had difficulties navigating the news, as they faced conflicting information on the issue. Naturally, the need to filter fake news becomes especially important when the news is significant for the person, their family and friends. Another central element of social representations is the knowledge that this social phenomenon affects everyone in society. Respondents seemed to believe that fake news is a danger to society and tended to distrust information initially, prior to verification. This was most typical for Generation Z (p = 0.000; CPA: 90.2, 90.3, 80.5 for Generation Z, “Millennials” and the Generation of Reforms, respectively). In general, fake news evoked mostly negative emotions (sadness, irritation, anger, a sense of inevitability, etc.) among respondents. As people of Generation Z and “Millennials” noted, conflicts often arise when discussing this issue with the older generation, since many parents believe in a kind of news that respondents deem obviously unreliable.

### Are there criteria for identifying the reliability of news information?

We have identified several key criteria for assessing the reliability of information, which are common core elements of social perceptions for all generations (see *Table 3*).

The criteria in *Table 3* include intentions to check information using several news sources, check official information, turn to primary sources, or read not only a brief overview of the situation but also follow the links provided to get better acquainted with the information. Respondents also considered their own knowledge and representations about various social situations, as well as their intuition and common sense when assessing news credibility. Checking news with their own representations helps people navigate the large volume of news and partition it into reliable and fake (unreliable) information. It was common for all generations to refer to the evidence provided in the news (quotes, videos, photos, links), but for younger generations this criterion was more important than for the Generation of Reforms (p = 0,000; CPA: 90,2, 90,3, 80,5 for Generation Z, Millennials and Generation of Reforms, respectively). Also, it was common for the three generations, especially “Millennials”, to assess the reputation and authority of the source, and pay attention to how the news is presented i.e., whether it is possible to distinguish the author’s opinion from fact. Participants negatively perceived the catchy, attention-grabbing headlines that are written in “marketing discourse”. Many “suspicious” advertisements that appeal largely to emotion make people doubt the reliability of the information. Generation Z was characterized by the belief that there are real ways to check the credibility of news oneself, whereas the Generation of Reforms was more prone to ask others — whether it be experts in a particular field, or people who are aware of and involved in the situation (between Generation Z and the Generation of Reforms: p = 0.029; between Generation Z and “Millennials”: p = 0.027; CPA: 71.5; 58.4 and 63.4). For example, during the focus group stage, several participants said that they were calling acquaintances in other countries to ensure that the news being broadcast in this country was true (about the “lockdown” or troubles during the coronavirus pandemic).

**Table 3:** The core elements of social representations for the block “criteria for the identification of the reliability of information in mass media”.

Statements	Generation Z (CPA)^a^	Millennials (CPA)	“Reformists” (CPA)
Statements included in the cores of all generations			
An important criterion for the reliability of the news is the evidence (links, quotes, photos, videos)	90.2	90.3	80.5
To assess the reliability of the news, I check the news using several sources	86.2	83.1	87.8
To define the reliability of the news, I go to the original source, the official source	85.4	85.1	85.4
The reputation and authority of the news source is an important criterion for me	77.2	87.0	81.3
I rely on my own understanding of the situation, on what I already know about the situation, when I define the reliability of the news	83.7	83.8	84.6

*Note. ^a^CPA= the coefficient of positive answers by J.Abric*.

### What factors shape my representations of the criteria for evaluating news information?

According to the participants, in addition to social institutions (parents, school, university), a significant factor is the personal experience of being confronted with fake information or making and analysing mistakes. The coverage of public events in Russia in the mass media has also impacted people’s attitudes towards certain sources of information. For example, the younger generations mentioned rallies and their conflicting coverage in different media (television vs. the internet). Participants tended to place more trust in sources that give information in full, without hiding facts, and provide photos and video evidence (mostly social media or the internet). The Generation of Reforms mentioned the period of the 90s, when, in their opinion, politicians often deceived the public, which led to a decrease in the level of trust in the media and government.

### What is the scope of false information spreading in the media now, and what is the forecast for the future?

In general, participants considered that there currently exists a lot of fake news, though percentage estimates differed (see *Table 4*). The most stable elements of the social representations are those associated with the understanding of fake news as a constant social phenomenon i.e., it existed before and will continue to exist forever. This view was especially pronounced among Generation Z (significant differences according to the Kruskal-Wallis criterion were between Generation Z and “Millennials”, p = 0.004; and between Generation Z and “Reformists” p = 0.000; CPA: 96.7, 93.5 and 95.9). Also, respondents believed that the quality and complexity of fake news will increase, making it harder to identify. This was a belief regarding which there were no significant generational differences.

**Table 4:** The core elements of social representations for the block “evaluation of the scope of false information spreading in the media in the past, present and the forecast for the future”

Statements	Generation Z (CPA)^a^	Millennials (CPA)	“Reformists” (CPA)
Statements, included in the cores of all generations			
Fake news has always been and will always be	96.7	93.5	95.9
Fake news will become more complex, better quality and more difficult to identify	76.4	72.1	78.9
There will be more and more unreliable news, as the internet and social networks will develop	47.2	53.2	53.7
Statements, included in the cores of 1–2 generations			
The amount of fake news used to be less than there is now	–	47.4	56.9
The amount of fake news will increase in the future	–	52.6	48.0

*Note. ^a^CPA= the coefficient of positive answers by J. Abric*.

The development of social media and the internet is one of the factors influencing this trend, as the total number of news sources and opportunities for news dissemination has and will continue to increase. Although the notion that there was less fake news in the past than there is now and will be in the future is quite stable, the forecasting capabilities of participants are still limited: they have difficulties with making unambiguous predictions.

### How do we solve the problem of the excessive prevalence of fake news?

In accord with the social representations of respondents, possible solutions to the fake news problem include improving the education system in the country, which will allow people to acquire information analysis skills and develop critical thinking. According to participants, uneducated people are more inclined to trust fake news. All generations considered it possible to influence the situation by helping their children navigate the large stream of information, teaching them how to filter and evaluate information, and nurturing them, so that they do not become the creators and distributors of fake news. As the participants also noted, the problem lies in the fact that many people are simply too lazy to check information and are quick to believe falsities (see *Table 5*).

**Table 5:** The core elements of social representations for the block “possible actions to solve the problem of fake news”.

Statements	Generation Z (CPA)^a^	Millennials (CPA)	“Reformists” (CPA)
Statements, included in the cores of all generations			
The main way to influence the presence of fake news is to improve the education system, educate people	89.4	88.3	89.4
The main way to reduce the amount of fake news is to influence our children, educate them morally and teach them critical thinking	81.3	93.5	91.1
If people weren’t too lazy to check the information, there would be fewer problems with fake news	82.1	85.1	83.7

*Note. ^a^CPA= the coefficient of positive answers by J. Abric*.

Younger generations perceived the situation as a problem that needs to be dealt with. Generation Z believed that special independent professional organizations should exercise control over the situation; and the difference between “Z” and “Reformists” was significant (CPA = 81.3, p = 0.008). Participants of all generations (representing the stable core element of social perceptions) considered that journalists should be given more freedom, so that they can visit event locations, communicate with eyewitnesses and find out the truth.

### How do trust, “trust in people” and representations about “fakes” correlate?

**Figure 3.**
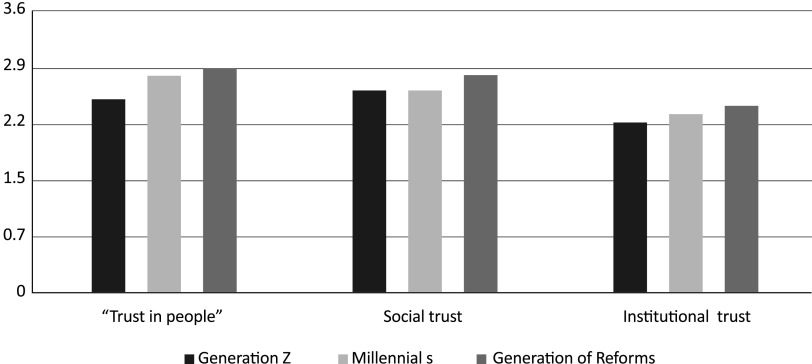
Measures of three types of trust.

*Note. The histogram illustrates the levels of basic trust in people, social trust, and institutional trust within the three generational groups*.

We measured the levels of basic trust in people, social trust, and institutional trust (see *Figure 3*). By all parameters, the average level of trust was low within this sample of respondents (most of the respondents scored between the 25th and 50th percentiles).

People from Generation Z had a lower level of “trust in people” (p = 0.00, Krus-kal-Wallis) than other generations. Respondents from the Generation of Reforms had a higher level of social and institutional trust (p = <0.00) than the younger generations. It was found that there are mostly negative correlations between the levels of trust and the core elements of social representations. A lower level of trust in people in general, society and social institutions is associated with the perception of fake news as a severe problem, behind which are representatives of social institutions or persons who deliberately seek to deceive people for their own gain (money, power, information warfare, etc.). If people do not trust other people, society, or social institutions, then they are more likely to perceive fake news as a danger and to believe that the media may broadcast fake information with the aim of deceiving or manipulating. In such situations, people are more likely to believe that politicians and authority figures may have something to do with it. High levels of trust in people will, on the contrary, contribute to trust in the creators of the news and create reliance on the author’s authority.

### What are the differences and similarities between generations in the social representations of “fakes”?

An important criterion for assessing news reliability for participants of all generations was relying on their own knowledge, representations of the situation, and intuition. People of different generations agreed that the main solutions to the problem are improving the education system in the country, educating their own children, avoiding laziness in information checking, and giving journalists more freedom to establish the truth. Younger generations were more likely to attribute fake news to accident, a mistake or incompetence on the part of the journalists, while “Reformists” mostly alluded to malicious intent of the creators of fake news. Generation Z were more inclined to perceive fake news as a habitual phenomenon for people and society, but this did not hinder their willingness to solve the problem. People of this generation were more convinced that there are certain ways to assess the reliability of information. Generation Z and “Millennials” focused on the presentation of information, the use of language, and headlines when identifying “fakes”. The Generation of Reforms tended to seek expert opinion or interact with people who are relevant to the situation.

## Discussion

### Social representations regarding “fakes”

1. The respondents we asked have **learned to live in a world of “fakes”.** Their understanding of “fakes”, representations about information reliability and its criteria, and the reasons for the appearance of inaccurate media facts correspond to theoretical studies of the phenomenon. Participants realize that “fakes” can be both intentional and accidental (due to journalistic faux pas), but they also often blame those with a vested interest in achieving selfish goals (striving for power, popularity, etc.). These effects are especially evident among respondents with low levels of trust. It is clear that: **the less I trust various social institutions, the more I must be on guard and the more it will seem to me that someone wants to deceive me deliberately.** This way of thinking allows a person to be in a state of heightened critical thinking. However, at peak levels of distrust, its carriers are approaching the a conspiracy theorist state (Egorova et al., 2020).

2. Participants are developing skills in extracting obviously unreliable information (for instance, everyone admits mistrusting the “marketing”, “overemotional”, “flashy” news presentation). Nevertheless, the strategies for masking fake information and deception in the media environment are developing and changing much faster than the audience can adapt to them. Thus, the respondents are confident in the need to educate media competence in society (courses in universities and schools), to stimulate the development of critical thinking and to bring up family correctly. It is also obvious that with the regularly changing design and form of “fakes”, a more important skill is discerning the content. An important element of the “core” is the representation of the necessity to increase freedom among journalists to reduce the number of “fakes”. Fake news prevalence as a result of a lack of journalistic freedom is an interesting conviction that deserves special attention.

3. “Fakes” turn out to be more significant the more they affect a person’s personality. This predictable result is vividly manifested in the context of the COVID-19 pandemic. Alarmingly for the majority of respondents, the disease has caused a large number “fakes” and discussions about them on social media (Egorova et al., 2020).

### Representations of “fakes” through the eyes of different generations

4. The representations of **Generation Z** stand apart: for them the presence of “fakes” in the media space is a habitual and familiar one. Their conscious strategy is associated with increasing verification of the surrounding information, so they use more fact-checking tools than other generations. At the same time, as mentioned above, fact-checking is not a clearly defined procedure: people can only assume that they can check information, due to the potential complexity of fact-checking and other obstacles such as value patterns.

5. At the same time, each generation demonstrates a certain way of dealing with “fakes”: if Generation Z uses digital verification (photos, videos, screenshots, correct quotes), then **“Reformists”** admit that they are prone to double-check the news through offline contacts with acquaintances and experts. Perhaps the pronounced propensity for fact-checking among Generation Z is just the outer shell, caused by a deeper knowledge of the environment of digital communication. Generation Z has come to terms with the reality of “fakes” and predicts that the amount of fake news will only increase as the media space becomes increasingly complex. Generation Z and **“Millennials”** have more core elements in their social perceptions of news reliability than the **Generation of Reforms.** It was expected that the Generation of Reforms rely on television more and on messengers less than Generation Z, which irritates the younger generation. The representation regarding the technological incompetence of the older generation in the eyes of the younger generation is reinforced by the less thorough fact-checking by older generations. Social representations of Generation Z and “Millennials” contain more negative core elements related to emotional attitudes towards fake news (the core element is the emotion of annoyance). In general, “Z” and “Millennials” are closer to each other in terms of representations than “Millennials” and “Reformists”. However, it should be remembered that comparing generations in relation to “fakes” can be problematic, especially when comparing different methods of information processing.

6. The **generation gap** with regards to “fakes” can be considered as a typical socio-psychological phenomenon: social perception manifests itself here in an explicit form, according to which we evaluate the age groups furthest from our own negatively. Thus, all generations consistently assess the media competence of people over 60 negatively, and at the same time, “Reformists” and “Z” negatively assess each other’s ability to recognize fakes. The fact that these two generational groups do not perceive “Millennials” in such a negative way only confirms the aforementioned claim, because millennials are a neighboring group for both older and younger generations. Also, both the “Reformists” and “Z” generations attribute high levels of media competence to their own groups. Mutual accusations of distant cohorts are a classic example of outgroup aggression, embedded within a typical intergenerational conflict.

7. It is curious that all generations consider that “news sites” are the least “fake” type of media. Generation Z is more prone to trust social networks as a source of information. The Generation of Reforms, as mentioned above, more often trusts TV news. At first glance, it seems that this evidence could be explained by the preference and familiarity of using certain media tools for older and younger participants. Nonetheless, this new hypothesis should be tested: *the fact that respondents tend to categorize information sources (television / internet / social networks), stereotypically and with overgeneralization, as more or less reliable, shows the vulnerability of these generational groups to potential fake information*. For example, if a respondent from Generation Z is convinced that television is more likely to deceive him than a telegram channel, he immediately becomes more vulnerable to unscrupulous communicators in modern social media, and vice versa.

## Conclusion

We have revealed the structure and content of social representations regarding the fake news and information reliability in the mass media and on social media among three generations of Russian people: the Generation of Reforms, the Millennial Generation and Generation Z. Upon comparison, we have found prominent differences between “Z” and “Reformists”, as well as similarities between “Z” and “Millennials”. Each generation demonstrated a certain way of identifying “fakes” (“Z” relies on fact-checking and digital verification; the Generation of Reforms checks the information through offline contacts with acquaintances and experts) and identified their preferred sources of reliable information (“Z” — social media, “Reformists” — TV).

Our findings provide evidence that “fakes” are purely social constructs: we tend to trust information that is consistent with our representations, beliefs, and attitudes. Moreover, people with rigid attitudes, regardless of their belonging to a particular generation, seem to be more vulnerable: if you understand that your knowledge is conditional, it is easier for you to live in a world of “fakes”; the more axiomatic ideas you have, the more problems you face with inaccurate information. In this respect, generations differ not in the quality of their fake news diagnostics, but in their tolerance towards “fakes”, and in their news verification strategies. The attribution of media incompetence to older and younger cohorts by each other is a typical case of the eternal conflict between “fathers and sons”.

The implications of these findings can be related to ways to improve media competence. Understanding the social representations of different generations regarding media information and reliability can help create a system for increasing media competence in a country, thereby preventing people from believing false facts and making decisions that harm themselves and society. Prevention and informational prophylaxis regarding fake news is more effective than dealing with the consequences after the fact (van Der Linden, Roosenbeek, Compton, 2020).

## Limitations

The collected data is based on self-report, so as stated, this research involved the study of representations, not actual behavior. The gender imbalance of the sample can be a possible limitation of our study (women prevail — 68% in the focus groups, 74.2% in the survey). This limitation was partially offset by the sample size at the survey stage (400 people). However, it is worthwhile to equalize the sample by gender in the future. Though the survey involved participants from different cities and regions of Russia (34 categories), participants from Moscow, the Moscow region and St. Petersburg made up the majority. The disproportion should be taken into account when interpreting the results, and in the future, we will increase the number of respondents from other regions of Russia, including small cities. These additions will allow us to extend the conclusions to the whole of Russia, and not only to the populations of large cities. We have also encountered the issue of distinguishing between age and generational features. This limitation can be overcome by conducting a longitudinal study.
